# Beyond Pathological Complete Response: Long-term Outcomes in Esophageal and Esophagogastric Junction Adenocarcinoma After Multimodal Therapy—A Systematic Review and Individual Patient-Data Meta-Analysis

**DOI:** 10.1245/s10434-026-20296-x

**Published:** 2026-07-27

**Authors:** Luca Risi, Renato De Martino, Lorenzo Giorgi, Paolo Enrico Meneghesso, Andrea Pansa, Silvia Basato, Carlo Castoro, Rita Alfieri, Giovanni Maria Garbarino

**Affiliations:** 1 Department of Biomedical Sciences, Humanitas University, via Rita Levi Montalcini 4, 20072 Pieve Emanuele, Milan Italy; 2 Upper Gastrointestinal Surgery Unit, IRCCS Humanitas Research Hospital, via Manzoni 56, 20089 Rozzano, Milan Italy

**Keywords:** Esophageal adenocarcinoma, Esophagogastric junction adenocarcinoma, Perioperative chemotherapy, Neoadjuvant chemoradiotherapy, Pathological complete response, Survival, Site of recurrence

## Abstract

**Background:**

Neoadjuvant chemotherapy (nCT) for esophageal and esophagogastric junction (EGJ) adenocarcinoma is associated with better survival but worse pathological complete response (pCR) rate as compared with neoadjuvant chemoradiotherapy (nCRT). The aim of the present study is to compare long-term outcomes in patients with esophageal or EGJ adenocarcinoma and pCR after nCT or nCRT.

**Patients and Methods:**

A systematic review of the literature in PubMed, Embase and web sources was performed up to November 2025. Studies comparing nCT and nCRT in patients with esophageal or EGJ adenocarcinoma and pCR were identified. An individual patient-data (IPD) meta-analysis was performed for overall survival (OS) and disease-free survival (DFS).

**Results:**

Overall, 1454 patients from six different studies were included (374 in nCT group and 1080 in nCRT group). 1-, 3-, and 5-year OS was superior in the nCT group as compared with nCRT group, being 95.4% versus 87.8%, 81.5% versus 64.8% and 73.8% versus 56%, respectively (*p* < 0.001). Analogously, superior DFS was observed for the nCT group at one, three and 5 years (*p* < 0.001). In the one-stage meta-analysis nCT was associated with decreased risk of death (HR = 0.49, *p* < 0.001) and recurrence (HR = 0.24, *p* <0.001).

**Conclusions:**

The present study showed better long-term outcomes in patients with pCR after nCT when compared with nCRT, highlighting the better systemic disease control offered by the first. This consideration suggests that pCR cannot be considered as a prognostic surrogate per se but the integration of this concept with the type of preoperative strategy is crucial.

**Supplementary Information:**

The online version contains supplementary material available at https://doi.org/10.1245/s10434-026-20296-x.

Esophageal and esophagogastric junction (EGJ) adenocarcinomas are aggressive malignancies, accounting for over 60% of esophageal cancers in Western countries, with a stable rising incidence rate.^1–3^ The combination of neoadjuvant chemotherapy (nCT) with or without radiotherapy (nCRT) before curative surgery, has significantly improved the survival of these patients,^4–6^ however, their prognosis is still poor, with a 5-year survival rate of only 20%.^2^

The CROSS-trial showed a survival benefit for chemoradiotherapy group followed by surgery when compared with surgery alone.^7^ Similarly, better overall survival was shown for patients undergoing nCT as compared with those who underwent upfront surgery.^6^ In particular, the FLOT-4 trials showed a survival benefit of chemotherapy on the basis of 5-fluorouracil, leucovorin, oxaliplatin, and docetaxel (FLOT scheme) over ECF/ECX chemotherapy (epirubicin and cisplatin plus 5-fluorouracil or capecitabine), becoming the standard scheme when feasible.^8,9^

Therefore, modern standards favor perioperative FLOT chemotherapy or neoadjuvant CROSS chemoradiotherapy.^9^ Although previous studies comparing preoperative chemotherapy and chemoradiotherapy showed no differences in long-term outcomes,^10^ the recent ESOPEC trial emphasized the superiority of FLOT over CROSS scheme.^11^ Choosing between perioperative FLOT and preoperative CROSS in locally advanced resectable adenocarcinomas is still debated but current international guidelines are in favor of perioperative FLOT.^12^

Pathological complete response (pCR) after neoadjuvant therapy is defined as ypT0N0 and it positively impacts long-term survival resulting in 49–66 months median overall survival (OS) and suggesting its role as a significant prognostic factor.^13,14^ When compared with CROSS, FLOT regimen showed significant lower pCR rates,^15^ but whether the neoadjuvant approach influences long-term outcomes in patients with pCR is still unclear. Retrospective studies report conflicting results, emphasizing the need to take into account factors beyond pCR, such as systemic disease control and recurrence pattern, with the neoadjuvant strategy possibly playing a role.^16–18^

The aim of the present study is to synthesize the existing literature on neoadjuvant treatments for esophageal and EGJ adenocarcinoma with a specific focus on comparing preoperative chemoradiotherapy and neoadjuvant chemotherapy to assess whether they can influence long-term outcomes, such as OS, disease free-survival (DFS) and pattern of recurrence, in patients with pCR.

## Patients and Methods

The current study aims to answer the following question: in patients with esophageal or esophagogastric junction adenocarcinoma and pathological complete response after multimodality treatment, is neoadjuvant chemotherapy associated with better long-term outcomes than neoadjuvant chemoradiotherapy?

In detail a PICOS was formulated: P (Patients)—patients with esophageal or EGJ adenocarcinoma undergoing neoadjuvant treatment and curative-intent surgery; I (Intervention)—neoadjuvant chemotherapy; C (Comparison)—neoadjuvant chemoradiotherapy; O (Outcomes)—primary outcome, overall survival and secondary outcomes (1) disease-free survival and (2) site of recurrence; and S (Study design)—systematic review of the available original articles and individual patient-data meta-analysis.

### Literature Search

PRISMA guidelines were followed (checklist available as Supplementary Table 1). The research protocol was registered at the International Prospective Register of Systematic Reviews (http://www.crd.york.ac.uk/PROSPERO) with registration number CRD42025630149.

A systematic review of the literature was performed in PubMed, Embase, and web sources (Google Scholar). The following search query was used: “(Esophageal Neoplasms OR esophag* OR oesophag* OR gastroesophag* OR gastrooesophag* OR junction* OR siewert) AND (neoplas* OR cancer OR cancers OR tumor* OR malign* OR carcinom* OR adenocarcinom* OR adc) AND (radio* OR drug* OR chemo* OR radiat* OR therap* OR treatment OR irradiat*) AND (preopera* OR pre-opera* OR adjuvant OR neoadjuvant OR neo-adjuvant OR peri-opera* OR periopera* OR pre-surg* OR presurg* OR CROSS OR FLOT OR Neoadjuvant Therapy) AND (pathologi* complete response OR pCR OR ypT0 OR ypT0N0 OR TRG 1a OR TRG 1 OR ypT0N0M0) AND (DFS OR disease free survival OR recurrence time OR recurrence free survival OR progression free survival OR RFS OR PFS OR recurrence rate OR early recurrence OR recurrence OR survival).” The search was restricted to studies involving humans and only English articles. No additional filters for type of study, date of publication or publication status were applied. The last search was run in November 2025.

### Study Selection

The papers identified through the electronic search were screened using the Rayyan platform (www.rayyan.ai)^19^. The software automatically detected duplicates, but they have been manually removed after being controlled by the authors. No further automatic support was used. After removal of duplicates, the titles and abstracts of the remaining articles were screened independently by at least two authors (LG, PEM, RDM, and LR). In the case of conflict, the final decision was taken by an additional independent evaluation (GMG). Subsequently, the full text of the selected articles was retrieved and read in detail to confirm eligibility. Finally, the reference list of the included articles was screened for additional studies.

The study included original articles comparing patients with esophageal or EGJ adenocarcinoma and pCR after neoadjuvant chemoradiotherapy or chemotherapy, and surgical complete resection. Randomized controlled trials, cohort studies, and case-control studies were considered eligible for inclusion. The following exclusion criteria were set: comparative studies including less than 10 patients, case reports, book chapters, editorials, previously published reviews and meta-analyses, conference proceedings, and consensus conference abstracts. In the case of multiple publications from the same research group or institution with significant overlap in terms of population and enrollment period, only the study with the largest cohort has been included.

FLOT is defined as a combination of fluorouracil, leucovorin, oxaliplatin, and docetaxel, MAGIC included cisplatin, fluorouracil, and epirubicin, OEO2 is composed of cisplatin and fluorouracil and CROSS is the combination of chemotherapy (carboplatin and paclitaxel) and concurrent radiotherapy.

### Data Extraction

The following data were extracted: (1) study characteristics, article metrics (i.e., name of the first author, institution of the corresponding author, year of publication, journal), study design and inclusion criteria; (2) cohort characteristics, i.e., number of patients, age, sex, body mass index (BMI), performance status [either defined as American Society of Anesthesiologists (ASA) score, Eastern Cooperative Oncology Group (ECOG) score, or Charlson Comorbidity Index (CCI)]; (3) adenocarcinoma data, i.e., tumor location, cT, and cN; (4) chemotherapy and chemoradiotherapy regimen; (5) intraoperative data, i.e., type of surgery, minimally invasive approach (laparoscopic, robotic, or hybrid); (6) pathology data of the tumor, i.e., grading, lympho-vascular invasion, perineural invasion, surgical margin; and (7) long-term outcomes, i.e., OS, DFS, and recurrence site.

All relevant texts, tables, and figures, including supplementary material were reviewed for data extraction. Data were independently extracted from the selected articles by two authors and then cross-checked (RDM and LR). Discrepancies between reviewers were solved by consensus discussion.

### Statistical Analysis

Continuous variables were reported as medians with interquartile ranges (IQR) or means with standard deviation whilst categorical variables as numbers and percentages. For survival outcomes, an individual patient-data (IPD) meta-analysis was performed, allowing computation of pooled survival estimates for each treatment group (nCRT and nCT), and comparison of pooled survival results between them. Survival data were extracted from published Kaplan–Meier curves using DigitizeIt software. For patient-level survival data reconstruction, the iterative algorithm by Guyot et al. was applied, estimating time-to-event parameters while maintaining the monotonicity constraint.^20–22^ For each study, the reconstructed survival data were reviewed by one author (LR) by comparing them with the original published data, including at-risk patient numbers, 1–5-year overall survival (OS) and disease-free survival (DFS) rates, log-rank and hazard ratio (HR) values, when available. A one-stage meta-analysis was conducted using Cox proportional hazards models with a gamma shared frailty model to account for dissimilarities in baseline rates among participants or groups owing to unmeasured covariates.^23,24^ The proportional hazards assumption was assessed visually by comparing predicted versus observed survival functions, and analytically by examining scaled Schoenfeld residuals and performing the Grambsch–Therneau test. Finally, as a nonparametric method of survival comparison the difference and ratio in restricted mean survival time (RMST) between groups were estimated by the naïve Kaplan–Meier method.^25,26^

*P*-values less than 0.05 were considered statistically significant for all tests. Statistical analysis was conducted using STATA version SE 19 (StataCorp, College Station, Texas, USA).

### Quality Assessment

The Newcastle-Ottawa Scale (NOS scale) was used to assess the quality of the comparative articles by two independent authors (RDM and LR)^27^. In case of discordance, a third author was involved to reach consensus.

## Results

**Fig. 1:**
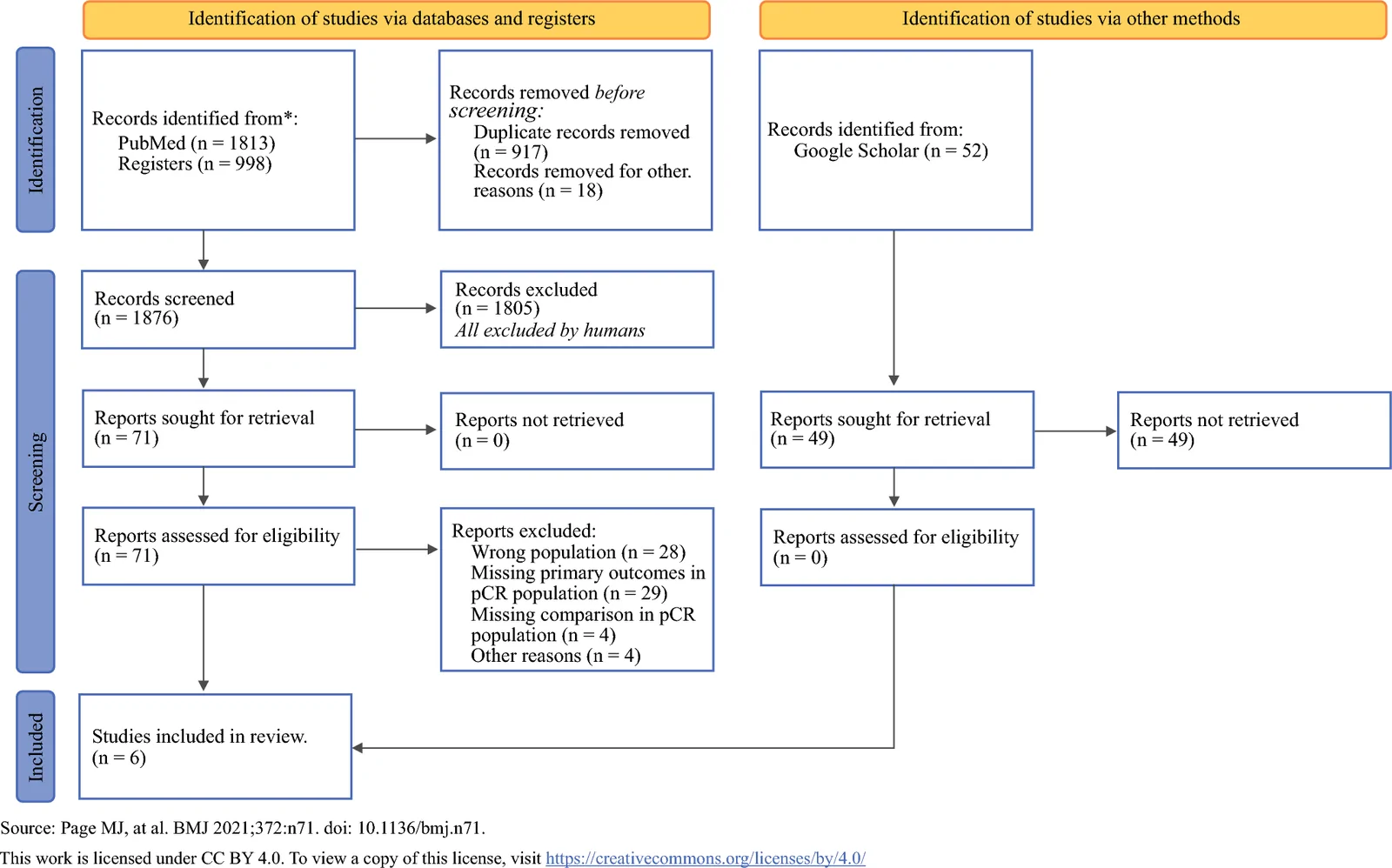
PRISMA flowchart

Figure 1 summarizes the selection process through the PRISMA flowchart. After the exclusion of duplicates and nonEnglish articles, 2863 papers were identified through the electronic search. Following screening, 71 full-text articles were eligible for retrieval: 28 articles were excluded for wrong population, 29 for missing long-term data in pCR population, 4 for missing comparison between nCT and nCRT in pCR population, and 4 for other reasons. Finally, six articles were considered eligible for the review.^16–18,28–30^

### Studies and Patients’ Characteristics

The main features of the included studies are listed in Table 1. All studies are retrospective and have been published between 2020 and 2025. All studies included patients from Western countries. Two studies are monocentric, with patients from German centers,^17,28^ and two are multicentric: one includes European institutions^18^ and one includes North American and European centers.^16^ Finally, two studies extracted patient data from the National Cancer Database which included patients from institutions in the USA with substantial differences in enrollment period and search query employed for patients enrollment.^29,30^ Considering all the included articles, the overall enrollment period spans from 2004 to 2021 with a median accrual time of 7 years (IQR, 7–9.25). Concerning only patients with pCR, all studies considered OS as the primary outcome, three included DFS (or RFS)^16–18^ and two included the pattern of recurrence too.^16,17^ Preoperative approach, inclusion criteria and definition of pCR for each study are presented in Table 2. Two studies did not properly detail the type of neoadjuvant chemotherapy and chemoradiotherapy,^29,30^ one considered mostly FLOT and CROSS as neoadjuvant approach,^18^ three considered only CROSS for chemoradiotherapy approach,^16,17,28^ two included only FLOT as chemotherapy regimen^17,28^ and one included FLOT, MAGIC and OEO2.^16^ All studies except one^29^ included esophageal adenocarcinoma, five out of six included esophagogastric junction cancer^16–18,28,29^ and two of them considered both esophageal squamous cell cancer (SCC) and adenocarcinoma but a subgroup analysis was performed exclusively on patients with adenocarcinoma.^17,28^ Only the study by Cools-Lartigue et al. was restricted to patients with pCR after neoadjuvant therapy.^16^ Hipp et al. included only patients with ypT0 despite the lymph nodes pathological status, however specific data about patients in the ypT0N0 group (pCR cohort) were available and then considered for the present analysis.^17^ Four studies defined pCR as ypT0N0,^16,17,28,29^ and, between them, one also included ypTisN0 in the definition.^29^ In one study, pathological response was quantified by the Mandard’s Tumor Regression Grade (TRG)^31^ with pCR being TRG1 and pN0.^18^ Only one study did not state the definition of pCR.^30^

**Table 1:** Metrics and characteristics of included articles

	Year	Journal	Country	Multicentric	Enrollment period	Long-term outcomes analyzed in pCR cohort
Capovilla G et al.^18^	2025	Annals of Surgical Oncology	Italy	Yes	2015–2020	OS, DFS
Cools-Lartigue J et al.^16^	2022	Annals of Surgery	Canada	Yes	2008–2018	OS, DFS, pattern of recurrence
Damanakis AI et al.^28^	2023	British Journal of Cancer	Germany	No	2012–2019	OS
Hipp J et al.^17^	2023	Langenbeck’s Archives of Surgery	Germany	No	2014–2021	OS, DFS, pattern of recurrence
Shridhar R et al.^30^	2025	Annals of Surgical Oncology	USA	NCDB*	2010–2017	OS
Xiang M et al.^29^	2020	Gastric Cancer	USA	NCDB*	2004–2014	OS

*OS* overall survival, *DFS* disease-free survival

^*^Data from National Cancer Database (NCDB)

**Table 2:** Adopted protocols, inclusion criteria, and definition of pathological complete response from included articles

	nCT scheme	nCRT scheme	Inclusion criteria	Definition of pCR
Capovilla G et al.^18^	Mostly FLOT	Mostly CROSS	Esophageal or EGJ (Siewert I-II) adenocarcinoma undergoing neoadjuvant treatment and radical surgery	TRG1 pN0
Cools-Lartigue J et al.^16^	MAGIC, OEO2, FLOT	CROSS	Esophageal or EGJ (Siewert I-II) adenocarcinoma with pCR after neoadjuvant treatment	ypT0N0
Damanakis AI et al.^28^	FLOT	CROSS	Esophageal adenocarcinoma or squamous cell cancer undergoing esophagectomy after CROSS or FLOT neoadjuvant therapy*	ypT0N0
Hipp J et al.^17^	FLOT	CROSS	Esophageal adenocarcinoma or squamous cell cancer undergoing multimodality treatment with curative intent with proof of post-operative pathological regression grade 1a according to Becker et al.*	ypT0N0ycM0
Shridhar R et al.^30^	Not stated	Not detailed	Esophageal adenocarcinoma treated with neoadjuvant chemotherapy or chemoradiotherapy undergoing esophagectomy	Not stated
Xiang M et al.^29^	Not stated	Not stated	EGJ adenocarcinoma undergoing surgery within 180 days from diagnosis after chemotherapy with or without radiotherapy	ypT0N0 or ypTisN0

*nCT* neoadjuvant chemotherapy, *nCRT* neoadjuvant chemoradiotherapy, *pCR* pathological complete response, *EGJ* esophagogastric junction, *TRG1* Tumor Regression Grade 1 (according to Mandard^31^)

^*^Only data about adenocarcinoma were considered for the analysis

Overall, 1454 patients were included in this study (374 in nCT group and 1080 in nCRT group) with a median study sample size of 215 (IQR, 102–392), 43 (IQR, 33–90) for the nCT group and 152 (IQR, 73–306) for the nCRT group. The available patient characteristics for each study are presented in Supplementary Table 2. Only two studies reported patient characteristics according to the neoadjuvant strategy and selectively for the pCR group.^16,17^

### Overall Survival Analysis

All studies provided 1-,3-, and 5-year OS rates. Four out of six studies showed a significant increase of OS in patients with pCR after neoadjuvant chemotherapy compared with chemoradiotherapy (*p* < 0.05 in all studies).^17,18,29,30^ By contrast, Cools-Lartigue et al. did not find a significant difference between the two groups with 5-year OS being 78.8% for perioperative chemotherapy group and 65.5% for neoadjuvant chemoradiotherapy group (*p* = 0.099).^16^ Different results were also found by Damanakis et al. showing lower 1-,3-, and 5-year OS rates for FLOT compared with CROSS.^28^

Five out of six studies reporting survival outcomes were included in the IPD meta-analysis. One study^16^ was excluded from the meta-analysis because it did not provide either the number at risk or the number of events.

**Fig. 2:**
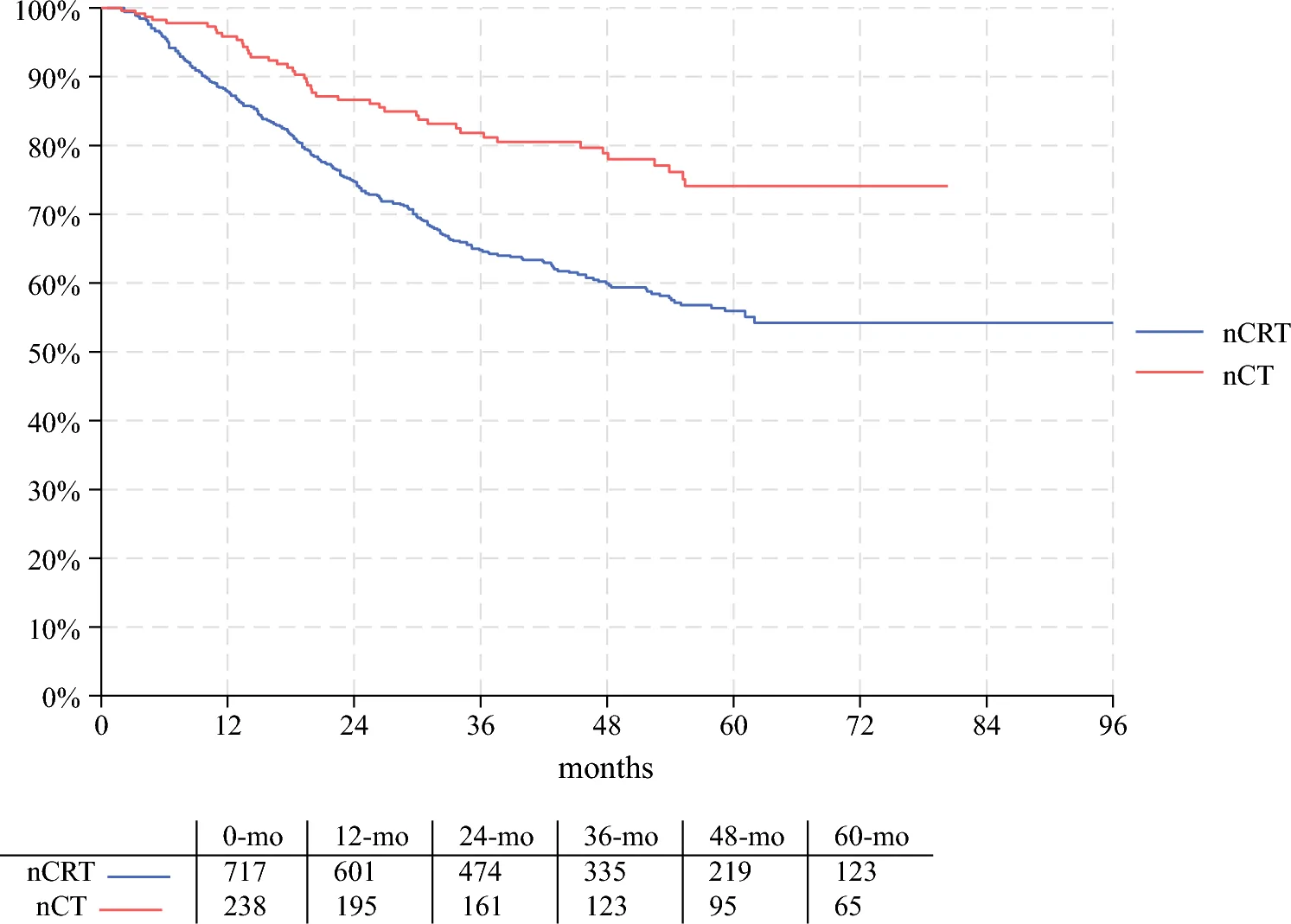
Pooled Kaplan–Meier for OS in nCT and nCRT groups

After the IPD meta-analysis the nCT group (*n* = 238) had 1-, 3-, and 5-year OS rates of 95.4% (95%CI 91.7–97.5%), 81.5% (95%CI 75.2–86.4%), and 73.8% (95%CI 66.1–80.1%), respectively. Rates were significantly lower in the nCRT group (*n* = 717), being 87.8% (95%CI 85.2–90%) for 1-year OS, 64.8% (95%CI 60.9–68.4%) for 3-year OS, and 56% (95%CI 51.5–60.2%) for 5-year OS (*p* < 0.0001) (Fig. 2). In the one-stage meta-analysis using Cox proportional hazards models, nCT was associated with significantly improved OS as compared with nCRT (HR = 0.49, 95%CI 0.36–0.68; *p* < 0.001). RMST values were 51 (95%CI 49–53.7) and 44 (95%CI 42.4–45.6) months for nCT and nCRT, respectively; in particular, nCT was associated with a significant increase of 7.4 months (95%CI 4.6–10.2 months; *p* < 0.001) of RMST at 5 years, which translates into a relative increase of life expectancy of 16.8% (95%CI 10.2–23.8%, *p* < 0.001).

### Disease-Free Survival Analysis

Three studies reported DFS data. Overall, DFS rates were superior for patients undergoing nCT compared with nCRT in all studies analyzing the outcome.^16–18^ In particular, in the study by Cools-Lartigue et al., nCRT was independently associated with poorer 5-year DFS as compared with nCT in the multivariate analysis [HR 1.70 (95%CI 1.22–2.99); *p*-value = 0.031].^16^

Two studies were included in the IPD meta-analysis.^17,18^ One study ^16^ did not provide either the number at risk or the number of events and it was excluded.

**Fig. 3:**
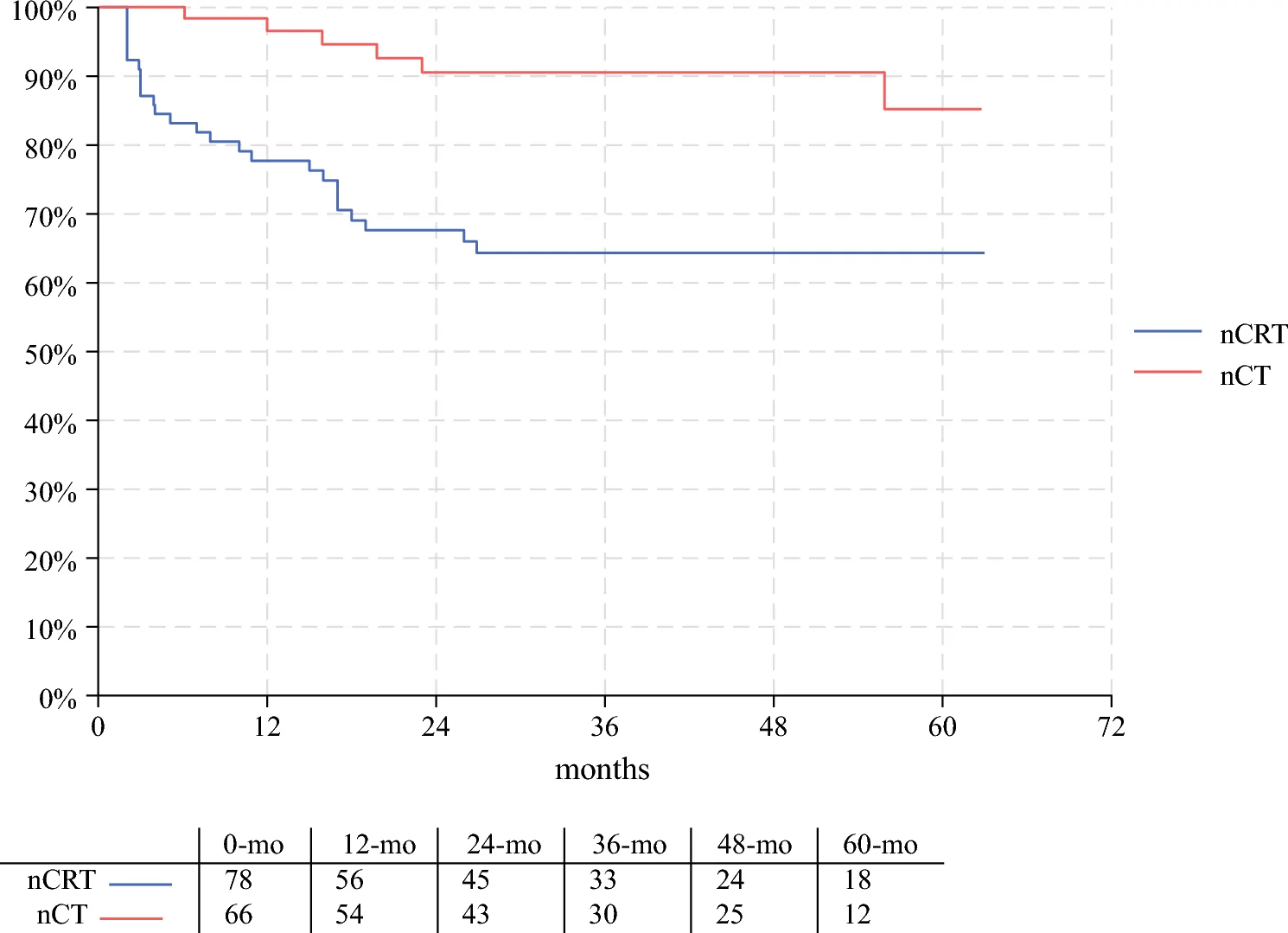
Pooled Kaplan–Meier for DFS in nCT and nCRT groups

In the IPD meta-analysis, 1-, 3-, and 5-year DFS rates for nCT group (*n* = 66) were 96.5% (95%CI 86.8–99.1%), 90.5% (95%CI 78.6–96%) and 85.2% (95%CI 66.8–93.8%), respectively. After nCRT (*n* = 78), 1-year DFS was 77.7% (95%CI 66.6–85.5%), 3- and 5-year DFS were 64.3% (95%CI 52–74.2%). DFS was significantly higher after nCT as compared with nCRT (*p* < 0.001) (Fig. 3).

In the one-stage meta-analysis using Cox proportional hazards models, nCT was associated with significantly improved DFS when compared with nCRT (HR = 0.24, 95%CI 0.1–0.59; *p* = 0.002). RMST were then estimated, being 55.6 (95%CI 52.1–59.1) and 42.2 (95%CI 36.6–47.7) months for nCT and nCRT, respectively. A significant increase of 13.5 months (95%CI 6.9–20.1 months; *p* < 0.001) of RMST at 5 years was found for patients undergoing nCT, which translates into a relative increase of disease-free life expectancy of 32% (95%CI 14–53%, *p* < 0.001).

### Pattern of Recurrence

Pattern of recurrence in patients with pCR was considered by two studies,^16,17^ summary data are presented in Supplementary Table 3. A meta-analysis of aggregated data was not performed due to scarcity and high heterogeneity of data. Cools-Lartigue et al. showed in the univariate analysis that pCR following nCT was associated with a significantly lower rate of 5-year distant recurrence when compared with nCRT (8.3% versus 22.5%, *p* < 0.001). The multivariate analysis confirmed the result with HR being 2.50 (95%-CI 1.24–4.99, *p*-value = 0.009). Considering the local recurrence, nCT showed worse results with 5.3% of 5-year recurrence rate, compared with nCRT (3%).^16^ In the study by Hipp et al., site of failure after CROSS was more frequently a distant recurrence in patients with esophageal adenocarcinoma and pCR. The cumulative incidence of distant recurrence (with or without concurrent local recurrence) in patients with pCR after CROSS protocol was significantly higher than patients after FLOT protocol [49% (±20%) versus 3% (±3%), *p* < 0.001; HR 0.041 (95%CI 0.004–0.406)].^17^

### Quality Assessment

According to the Newcastle Ottawa Scale, the included studies had an overall good quality with 3 articles achieving a score of 9 and 3 a score of 8 (Supplementary Table 4).

## Discussion

The present study collected a significant number of patients with esophageal or EGJ adenocarcinoma and pCR after multimodality therapy, showing better OS and DFS after nCT, implying a better control of the distant disease and potentially challenging the paradigm of pCR as a prognostic surrogate.

Pathological complete response is known as an important prognostic factor, positively impacting on long-term outcomes with increased overall survival and disease-free survival when compared with partial or absent pathological response.^32,33^ This evidence led us to consider the pCR as a valid oncological surrogate in predicting a favorable prognosis.

As previously demonstrated, nCT has a lower pCR rate,^11^ leading to the hypothesis of worse survival outcome as compared with nCRT and potentially encouraging the use of nCRT.

Superiority of nCT over nCRT remained uncertain until the recent randomized clinical ESOPEC trial showed better outcomes for patients undergoing FLOT compared with CROSS, despite the lower pCR rate in the FLOT cohort, prompting reconsideration of whether FLOT should replace CROSS regimen as the gold standard of multimodality treatment for resectable esophageal or EGJ adenocarcinoma.^10,11,34,35^

However, the efficacy described in clinical trials often fails to translate into similar effectiveness in everyday clinical practice. Previous real-world studies confronting long-term outcomes of patients undergoing FLOT or CROSS protocol failed to show the superiority of the first one over the latter^36,37^ but they raised important considerations about results in clinical trials. In the real-world study by van Laarhoven et al. concerning patients with esophageal and EGJ adenocarcinoma undergoing CROSS regimen, substantial differences with results in the ESOPEC trial emerged. Firstly, completion rate of nCRT in the study was similar to the one reported in CROSS trial (88% and 91%, respectively) but higher than the one in ESOPEC trial (75%).^7,11,14^ Analogously, pCR rate in the study and in the CROSS trial were comparable (20% and 23%, respectively) but substantially higher than the one in the ESOPEC trial (10.1%).^7,11,14^ Substantial differences were also seen when comparing severe adverse event rate (grade ≥ 3) after nCRT in CROSS and ESOPEC trial (50% versus 23%).^7,11^ This evidence led to the hypothesis that the management of chemoradiotherapy in ESOPEC trial was suboptimal as compared with standard clinical practice, possibly impairing outcomes for the nCRT cohort. Therefore, the dissimilarities between clinical trials and real-world results may have raised doubts about real superiority of FLOT over CROSS regimen, leading the clinicians to still consider the role of CROSS protocol in clinical practice. As a matter of fact, one major concern about abandoning nCRT is the higher pCR rate achieved and the strong connection of pCR with a favorable prognosis.

Nevertheless, the discrepancy between the optimal survival results after nCT and its lower pCR rate leads to an open question: may the conviction of pCR as a prognostic surrogate acquire a different meaning according to the neoadjuvant approach?

Trying to answer this main question, the present study challenged the paradigm of pCR as a prognostic surrogate per se. Different survival results according to the neoadjuvant strategy were reported, with nCT showing better outcomes, probably owing to better control of the distant disease.

These findings advocate the hypothesis that the local tumor response cannot be exclusively considered an optimal predictor of better survival and pCR cannot be supposed as a prognostic factor per se, but it needs to be correlated with the neoadjuvant strategy.

The failure of nCRT in translating into an improvement of long-term outcomes, despite its higher pCR rate,^11^ may be owing to a worse control of the distant disease. In the study by Capovilla G et al. patients were stratified by tumor regression grade and pathological nodal status and, despite the higher rate of pCR in the nCRT group, tumor regression grade was associated with better OS and DFS only after nCT, independently of the pathological nodal status, confirming that an improved local tumor response cannot be considered a predictor of better survival in the nCRT group.^18^

In patients undergoing nCRT, the higher pCR rate might be a consequence of the direct effect of the combination of radiation and chemotherapy on the tumor. On the contrary, the perioperative chemotherapy has a systemic effect, possibly achieving better control of the distant disease as compared with chemotherapy in CROSS regimen which is more radiosensitizing.^38^ This hypothesis is strongly suggested by the higher incidence rate of distant recurrence in patients with pCR after neoadjuvant chemoradiotherapy, as described by Cools-Lartigue et al. and Hipp et al.^16,17^ Therefore, better control of the distant disease might translate into better long-term outcomes, possibly explaining the superiority of nCT over nCRT.

To overcome these limits, combining the local effectiveness of chemoradiotherapy and the systemic effect of chemotherapy, the RACE trial is currently investigating the possibility of preoperative induction chemotherapy followed by preoperative chemoradiotherapy for esophageal cancer (NCT04375605).^39^

The introduction of combined chemotherapy and immunotherapy in a neoadjuvant setting could ulteriorly change perspectives. Trials such as DANTE and MATTERHORN have demonstrated that the addition of anti-PD-L1 immune checkpoint inhibitors to perioperative FLOT significantly enhances pCR rates. However, a subgroup analysis comparing survival outcomes and recurrence pattern in patients having pCR was not reported, leaving an open debate concerning the role of pCR after neoadjuvant immunochemotherapy.^40,41^

For patients with HER2-positive esophageal or EGJ adenocarcinoma, although the PETRARCA trial showed improved pCR rates with dual HER2 blockade, the INNOVATION trial cautioned that pathological regression is an unreliable surrogate for survival; nevertheless, the recent phase II PHERFLOT trial successfully explored an intensified triple-modality regimen combining FLOT, pembrolizumab, and trastuzumab, achieving an exceptional intention-to-treat pCR rate of 48.4% with a feasible safety profile.^42–44^

Local and distant recurrence after pCR can still occur but until now the reasons behind it remain unknown. Micrometastatic disease and tumor biology may play a role, with tumor differentiation being an independent prognostic factor for recurrence in patients with esophageal cancer with pCR.^32,45^ Esophageal adenocarcinoma is heterogeneous in molecular and biological features, and this may lead to unpredictable response to neoadjuvant treatments, which translates into different changes in microscopical aspect of the specimen after neoadjuvant chemotherapy or chemoradiotherapy.^31,46^ A recent study by Hoefnagel et al. showed a significant association between the tumor biological profile and tumor regression after neoadjuvant treatments supporting the idea of a biological foundation underlying the response to nCT and nCRT which may influence the probability of recurrence after pCR .^47^

Also, technical limitations of pathology evaluation must be taken into account with possible residual tumor being below the detection threshold and possibly leading to local and distant recurrences. In this scenario future studies involving liquid biopsy to detect any residual disease will probably help to understand these aspects, possibly changing the perception of pCR.^48^

Some limitations should be acknowledged when interpreting these results. Firstly, only a few articles were included, with high heterogeneous data, hampering the possibility to create stronger evidence. Secondly, only two out of six studies reported clinical characteristics of patients with pCR and limited data about tumor biology and type of surgery were available among the studies, limiting the possibilities to provide reliable summary and comparison, and potentially leading to misinterpretation of results. Thirdly, two studies did not detail the nCRT protocol, and the nCT regimens were not uniform with two studies not reporting further details. Differences in efficacy between chemotherapy schemes is well-known and they could be obscured by grouping the regimens together.^8^ Fourth, two studies included SCC patients and, although they performed an isolated subgroup analysis on adenocarcinoma patients, potential for confounding should be considered. Fifth, the cohorts’ size was unbalanced, with a greater median sample size of nCRT group, reflecting its higher pCR rate and possibly influencing comparisons. Finally, all patients were selected from institutions from Western countries. Despite esophageal cancer being more frequent in Asia, SCC is still the most common histologic subtype in Eastern countries whilst recently the incidence rate of esophageal adenocarcinoma rose in Western countries becoming the most frequent histologic subtype in Europe and North America in 2020.^49,50^ The combination of these trends and the search query used for this review selecting for “adenocarcinoma,” might explain this last consideration.

Nonetheless, the present review has some merits. Firstly, with rigorous methodology, it gathered all available studies estimating pooled long-term outcomes in patients with pCR and effectively comparing nCT and nCRT through an IPD meta-analysis. To the best of our knowledge, this is the first study trying this effort. Secondly, the present study presents some relevant methodological considerations. Although numerous articles confronting nCT with nCRT were available (71 articles assessed for eligibility), the majority of them mixed different histological subtypes (adenocarcinoma and SCC) or pCR with non-pCR patients, or they did not perform a subgroup analysis on patients with pCR. This may be owing to the lack of consistent data, especially in the nCT cohort, underscoring the need for further studies to collect specific data and produce focused evidence.

In conclusion, by acknowledging the limitations of the present review and meta-analysis, these findings might support the hypothesis that, in patients with pCR, nCT seems to offer better systemic disease control compared with nCRT, which translates into better survival outcomes. This consideration should lead the clinicians to discuss the validity of pCR as a prognostic surrogate per se, highlighting the need to look at it from a different perspective through the consideration of the type of preoperative strategy.

Finally, this evidence should further encourage the employment of neoadjuvant chemotherapy instead of chemoradiotherapy when deciding the type of neoadjuvant strategy for resectable esophageal or EGJ adenocarcinoma.

There is an urge for additional studies to better elucidate the prognosis of patients with pCR after nCT and nCRT, especially clarifying the role played by the pattern of recurrence after pCR and according to the neoadjuvant strategy. Additionally, new data on pCR assessment methods, such as the liquid biopsy, should be collected to further refine their contribution.

## Supplementary Information

Below is the link to the electronic supplementary material.

Supplementary file 1 (DOCX 3195 KB)

## Data Availability

The present data are available on reasonable request.
